# Case report: Severe, refractory hypoglycemia in a 9-year-old Brittany Spaniel with renal nephroblastoma

**DOI:** 10.3389/fvets.2023.1116846

**Published:** 2023-04-18

**Authors:** Paula Simons, Bridget Lyons, Adrienne Bentley, Elisa Mazzaferro, Lindsay Thalheim, Riccardo Finotello, Lorenzo Ressel

**Affiliations:** ^1^Department of Emergency and Critical Care, Cornell University Veterinary Specialists, Stamford, CT, United States; ^2^Department of Surgery, Cornell University Veterinary Specialists, Stamford, CT, United States; ^3^Department of Oncology, Cornell University Veterinary Specialists, Stamford, CT, United States; ^4^Department of Small Animal Clinical Science, Institute of Infection Veterinary and Ecological Sciences, University of Liverpool, Liverpool, United Kingdom; ^5^Department of Veterinary Anatomy Physiology and Pathology, Institute of Infection Veterinary and Ecological Sciences, University of Liverpool, Liverpool, United Kingdom

**Keywords:** hypoglycemia, nephroblastoma, non-islet cell tumor-induced hypoglycemia (NICTH), oncology, nephrectomy, glucagon

## Abstract

A 9-year-old female spayed Brittany Spaniel presented for weakness and stumbling, and was diagnosed with severe hypoglycemia. An insulin to glucose ratio was not consistent with insulinoma as a cause for hypoglycemia. Diagnostic imaging (abdominal ultrasound and computed tomography) revealed a large left renal mass and a possible metastatic lesion in the right kidney. Glucagon therapy was initiated, but hypoglycemia was refractory to therapy. A left nephrectomy was performed and hypoglycemia subsequently resolved. Histopathology of the mass was consistent with nephroblastoma and immunohistochemistry for anti-insulin-like Growth Factor-2 (IGF-2) antibody revealed immunoreactivity in over 50% of the neoplastic cells. Chemotherapeutic treatment was initiated with a combined protocol of vincristine and doxorubicin. To the authors' knowledge, this is the first case report documenting the treatment of severe, refractory non-islet cell tumor-induced hypoglycemia in a dog, suspected to be secondary to an IGF-2 secreting nephroblastoma.

## 1. Introduction

Primary renal tumors are rare in dogs and constitute 0.6%−1.7% of all neoplasms, with the majority being renal cell carcinomas (1, 2). Nephroblastoma comprises about 10% of primary renal tumors and is most commonly found in juvenile, large-breed dogs < 2 years of age (3). This malignant neoplasm is derived from nephrogenic rests, which are embryonic progenitor cells that are retained in the postnatal kidney and continue to proliferate (1). Clinical signs are non-specific and include polyuria, polydipsia, vomiting, weight loss, lethargy, and rarely, hypoglycemia. In humans, nephroblastoma (commonly referred to as Wilms' tumor) is the most common form of pediatric renal cancer (4). Treatment includes nephrectomy followed by systemic chemotherapy (13).

Paraneoplastic hypoglycemia is the hallmark of pancreatic beta-cell neuroendocrine tumors in dogs (5). Non-islet cell tumor-induced hypoglycemia (NICTH) has been reported in dogs with hepatocellular carcinoma, hepatoma, renal adenocarcinoma, leiomyoma, leiomyosarcoma, pulmonary adenocarcinoma, and mammary carcinoma (6–12). Children with Wilms' tumors have experienced IGF-2-induced NICTH but this sequela is not well described in veterinary patients (13). The pathophysiology of hypoglycemia in dogs with nephroblastoma has not been investigated, but rather speculated to be secondary to polycythemia in one case report (14, 15). This case report describes the treatment of refractory hypoglycemia in a dog diagnosed with nephroblastoma that resolved with nephrectomy. Immunohistochemistry revealed that 50% of the neoplastic cells were immunoreactive for IGF-2 supporting IGF-2 as a causative factor of severe hypoglycemia.

## 2. Case description

A nine-year-old, 17 kg, female spayed Brittany Spaniel was presented for an ~1-week history of disorientation, weakness, and stumbling. The dog had a prior history of degenerative mitral valve disease, and she had previously been treated for mammary carcinoma with initial diagnosis and surgical excision in 2018, as well as pulmonary adenocarcinoma with left radical mastectomy and right caudal lung lobectomy performed concurrently in 2020. There was no evidence of recurrence of either mammary nor pulmonary neoplasia based on screening abdominal ultrasound and thoracic radiographs performed at 3–6 month intervals following diagnosis and surgical excision. The dog was reportedly eating well, but was polydipsic and polyuric prior to presentation. No overt episodes of collapse or seizure activity were described by the owner. The patient's referring veterinarian had performed diagnostic testing 24 h prior to presentation that included a complete blood count (CBC), biochemical analysis, thyroid testing, urinalysis, vector-borne disease testing,^1^ and abdominal radiographs. The CBC was within normal limits and a serum biochemistry panel revealed moderate hypoglycemia (2.5 mmol/L; reference range 3.61–6.22 mmol/L). A total thyroxine (T4) test was within normal limits. Urinalysis showed hyposthenuria (urine specific gravity 1.006; reference range 1.015–1.050) and alkaline urine (pH 8; reference range 5.5–7.0). Vector-borne disease testing was negative for *Ehrlichia canis, E. ewingii, Anaplasma phagocytophilum, A. platys, Dirofilaria immitis*, and *Borrelia burgdorferi*. Abdominal radiographs revealed a mass effect in the region of the left kidney. On presentation to the referral hospital, physical examination revealed a grade II/VI systolic left apical murmur and a palpable mid-to-caudal abdominal mass.

The patient was neurologically appropriate, and the remainder of the physical examination was unremarkable.

**Figure 1:**
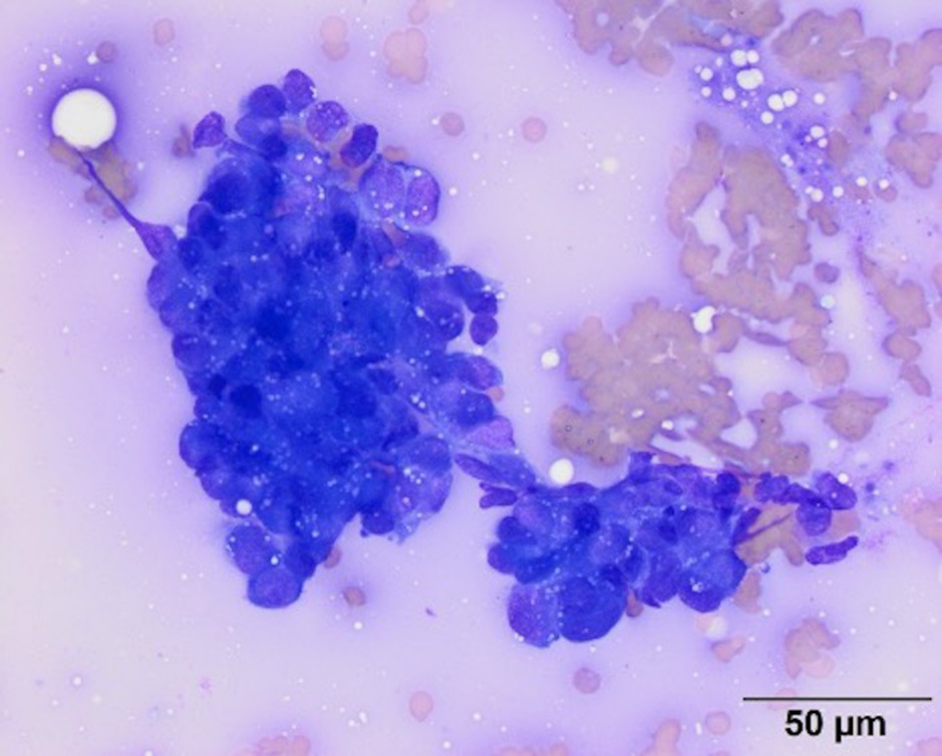
Representative image of a smear from a fine needle aspirate of the left renal mass. Cytologic examination of modified Wright's stained smears revealed moderate numbers of irregularly shaped dense clusters of medium to large (12–20 um) round to cuboidal cells. The cells had round to ovoid nuclei with fine chromatin and inapparent nucleoli. They had high nuclear to cytoplasmic ratios with small amounts of medium to dark blue cytoplasm with low numbers of discrete margined vacuoles. Based on the cohesive appearance, a cytologic diagnosis of carcinoma was made. Scale bar = 50 microns.

Point-of-care diagnostics at the time of presentation showed severe hypoglycemia (1.1 mmol/L; reference range 3.61–6.22 mmol/L), hyperlactatemia (2.41 mmol/L; reference range 0.5–2.0 mmol/L), and a base deficit (−5.5 mmol/L, reference range −5 to 1 mmol/L). Abdominal ultrasonography was performed and revealed a large (10.9 cm × 6 cm × 6.2 cm) moderately vascularized, bilobed mass arising from the caudolateral aspect of the left kidney with differentials to include nephroblastoma, renal sarcoma, and renal carcinoma. The mass was mildly heterogeneous with hyperechoic retroperitoneal fat surrounding the mass. The left renal pelvis was dilated at 0.8 cm (reference range <2mm). The right kidney appeared progressively enlarged from the patient's previous ultrasonographic exam with decreased corticomedullary definition. The right renal cortex was hyperechoic and thick with mild dilation of the renal pelvis and diverticulum. Right ureteral dilation was not detected. These changes were not apparent on abdominal ultrasound performed 5 months prior. An ultrasound-guided fine needle aspirate of the left renal mass was performed, and cytology was consistent with carcinoma as characterized by round to cuboidal epithelial cells that had high nuclear to cytoplasmic ratios with small amounts of medium to dark blue cytoplasm and low numbers of discrete margined vacuoles. The nuclei within the cells were noted to be round to ovoid with nuclear crowding in clusters (Figure 1). An insulin panel^2^ was submitted, which ultimately revealed hypoglycemia (1.33 mmol/L; reference range 3.5–6.33 mmol/L) and hypoinsulinemia (<2.0 uIU/ml; reference range 5.2–41.5 uIU/ml). These findings were not supportive of insulinoma as a cause of the dog's hypoglycemia. Based on the diagnostic results, renal neoplasia was suspected either as a primary neoplasm or secondary to metastasis of the patient's previously diagnosed pulmonary adenocarcinoma or mammary carcinoma.

During hospitalization, the patient remained markedly hypoglycemic despite having a good appetite and frequent feedings. A glucagon continuous rate infusion [50 ng/kg IV bolus followed by 10 ng/kg/min IV (CRI)] was initiated on the second day of hospitalization. The dog's blood glucose improved to 9.88 mmol/L (reference range 3.61–6.22 mmol/L) but again fell several hours later and was too low to measure (< 1.1 mmol/L). The glucagon CRI was titrated upward to 12 ng/kg/min with mild improvement in blood glucose concentration (2.5 mmol/L; reference range 3.61–6.22 mmol/L). Overnight, the glucagon CRI continued to be titrated upward to a maximum rate of 40 ng/kg/min due to persistent hypoglycemia. During the periods of hypoglycemia, the patient exhibited generalized weakness, was quiet but responsive, and had no seizure activity.

**Figure 2:**
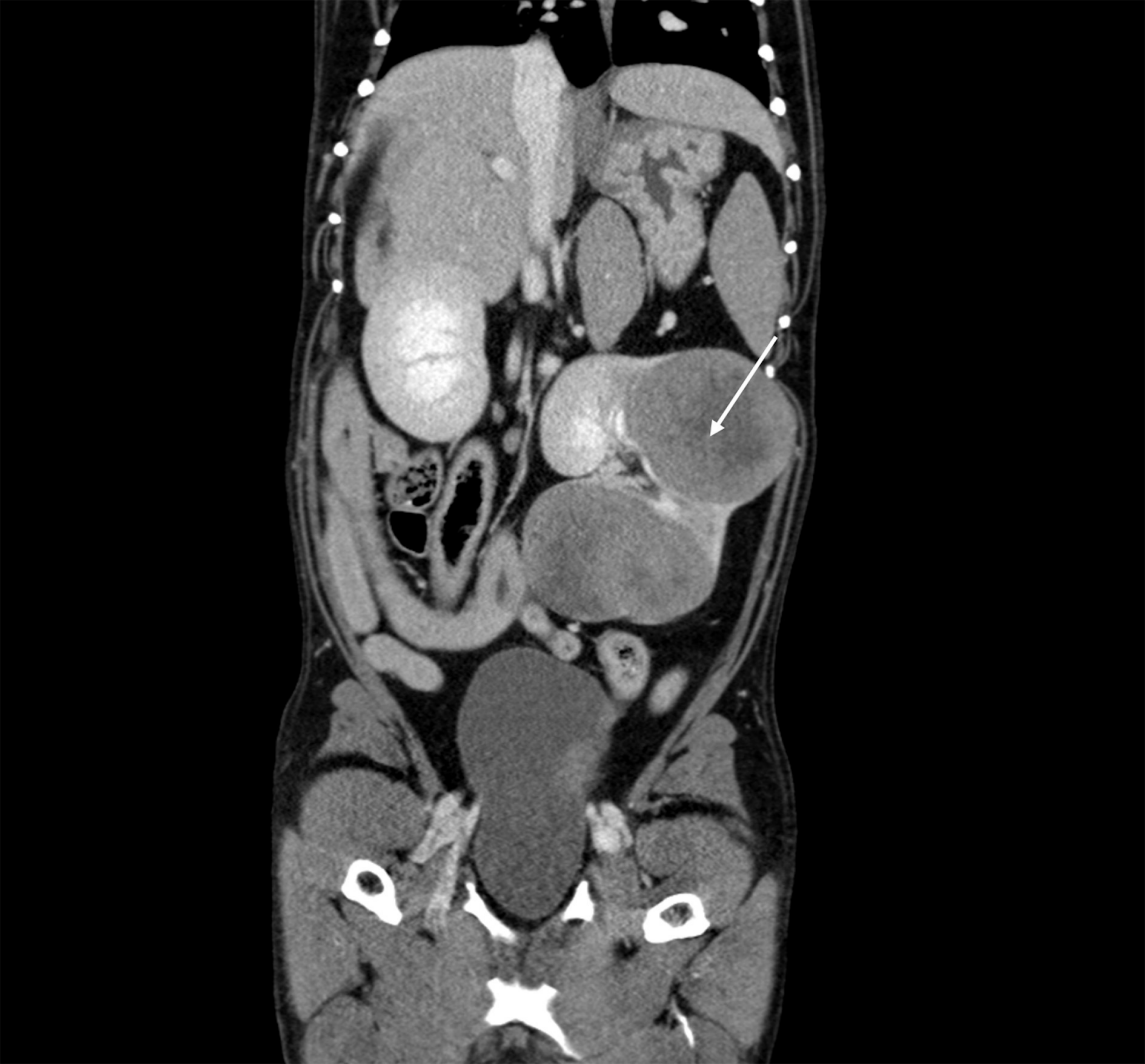
Computed tomography: coronal view of the abdomen with the caudal thorax at the top of the image, demonstrating a large, complex, multilobular mass arising from the left kidney (arrow).

On the third day of hospitalization, thoracic and abdominal computed tomography (CT) was performed. A large, complex, multilobular mass arising from the left kidney was found (Figure 2). The mass measured 10.9 cm in length by 9.1 cm in width by 7.2 cm in height at the largest dimension. This mass was hypoattenuating to the rest of the kidney, with the center of the mass being profoundly hypoattenuating after contrast administration. The mass was found to invade the diverticular recesses/renal pelvis of the left kidney, leading to mild renal pelvic dilation (0.7 cm). A hypoattenuating soft tissue lesion with similar CT characteristics to the left renal mass was noted in the medial aspect of the cranial pole of the right kidney. This lesion measured 2.7 cm × 1.8 cm at the largest cross-section. The liver was diffusely enlarged, with normal attenuation and contrast enhancement. The remainder of the abdomen was normal. On CT evaluation of the thorax, metallic hemostatic clips associated with the previous right caudal lung lobectomy site showed a minimal amount of unstructured increased soft tissue attenuation in this region of the lungs which was an expected postoperative finding. A small amount of increased soft tissue attenuation in the dorsal lungs bilaterally was attributed to atelectasis. No pulmonary soft tissue nodules were detected. Recurrence of pulmonary adenocarcinoma could not be excluded without histologic evaluation, though is considered unlikely.

The patient was premedicated with methadone (0.3 mg/kg IV) and dexmedetomidine (1 mcg/kg IV) and was then induced with propofol (2 mg/kg IV) and lidocaine (2 mg/kg IV) to effect. Following endotracheal intubation, the dog was maintained on gas anesthesia (1.5% isoflurane in oxygen). Lidocaine (30 mcg/kg/min titrated upward to 40 mcg/kg/min), dexmedetomidine (0.25 mcg/kg/h), and glucagon (starting rate of 40 ng/kg/min, increased to 50 ng/kg/min, then weaned to 20 ng/kg/min) CRIs were continued intraoperatively. A left nephrectomy was performed through a midline laparotomy to remove the large mass in the left kidney. The lesion in the cranial pole of the right kidney was palpable but was not sampled due to the risk of hemorrhage. After removal of the left kidney, the patient's blood glucose improved to 3.22 mmol/L (reference range 3.61–6.22 mmol/L). The glucagon CRI was weaned over several hours and discontinued when the patient became mildly hyperglycemic at 7.1 mmol/L (reference range 3.61–6.22 mmol/L) 3 h after surgery. The patient remained normoglycemic and was discharged 2 days following surgery.

**Figure 3:**
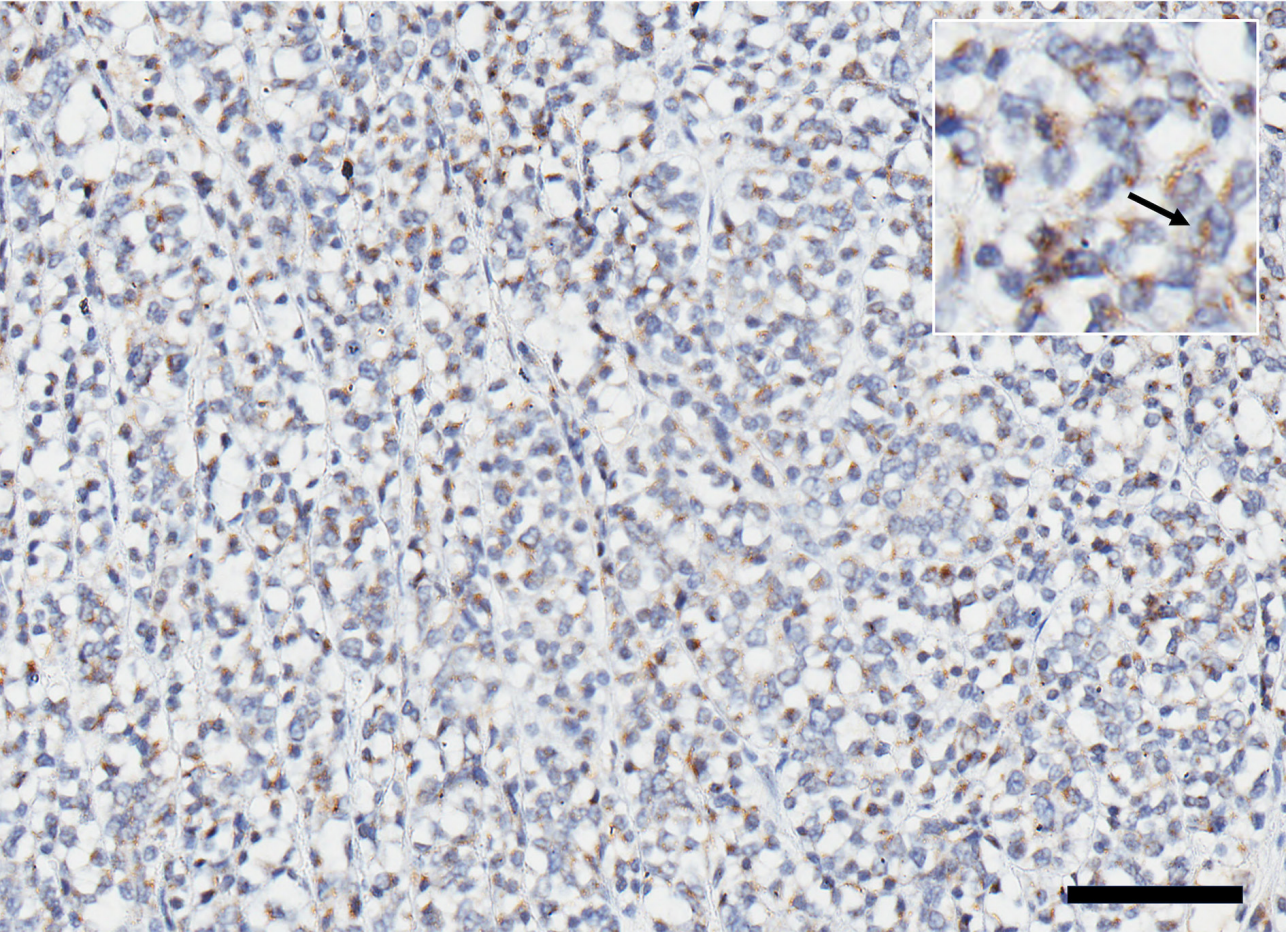
Expression of IGF 2 represented by immunoreactive brown stain in ~50% of the cells within the cytoplasm; **(inset)** higher magnification of positive cells (arrow). Scale bar = 200 microns.

Histopathological examination of the left kidney showed a multilobular infiltrative neoplastic proliferation of basaloid polygonal cells supported on a fibrovascular stroma arranged in tubules, nests, infoldings, and sheets. Morphologic features of the neoplasm were most suggestive of a nephroblastoma despite the lack of abundant primitive glomeruloid structures, given the presence of a solitary renal tumor which contained epithelial, blastemal, and mesenchymal populations. Only the glomeruloid variety of the epithelial population was not noted. The neoplastic cells had oval stippled nuclei, one to two magenta nucleoli, scant eosinophilic cytoplasm, and indistinct borders. There was moderate anisocytosis and anisokaryosis. A mitotic count of 18 in 2.37 mm^2^ was noted with no evidence of lymphovascular invasion. Immunohistochemistry staining performed on the mass was positive for vimentin in the mesenchymal cells, cytokeratin AE1/AE3 in the epithelium, and WT-1 (Wilms' tumor gene). The histomorphologic features combined with the immunohistochemical results were most supportive of nephroblastoma. Immunohistochemistry for IGF-2 revealed that more than 50% of the neoplastic cells were immunoreactive with diffuse cytoplasmic granular staining (Figure 3). The clinical findings, histology and immunohistochemistry findings together support IGF-2 production by the neoplasm.

A combination chemotherapy protocol with vincristine (0.6 mg/m^2^ IV) and doxorubicin (1 mg/kg IV) was initiated 13 days postoperatively with vincristine and doxorubicin being alternated every 2 weeks. Dactinomycin treatment was considered but not pursued due to cost and difficulty obtaining the chemotherapeutic agent. The dog has continued to do well clinically 6 months post-diagnosis of nephroblastoma, though the right renal lesion is visible on abdominal ultrasound interrogation (2.6 cm × 2.5 cm at largest cross-section). No recurrence of hypoglycemia was noted 6 months after surgery.

## 3. Discussion

This is the first case report of a dog with severe, refractory NICTH secondary to a nephroblastoma, with documentation of IGF-2 expression. Non-islet cell tumor induced hypoglycemia secondary to nephroblastoma has been reported in two other cases including a 17-month-old Jack Russell Terrier and a young female Beagle (14, 15). Hypoglycemia has been documented secondary to nephroblastoma but was considered to be secondary to polycythemia and was not as severe as the patient presented in this report (14). In a case report by Coleman et al., a young Beagle was described to have moderate hypoglycemia suspected secondary to embryonal nephroma but the cause was not further investigated (15). The presence of IGF-2 secretion was not evaluated in the aforementioned cases. Glucagon infusion was not utilized in either case to manage hypoglycemia. This patient's severe hypoglycemia was considered to be paraneoplastic in origin given the lack of a source of sepsis, polycythemia, hepatic dysfunction, or exogenous insulin administration, and with resolution following nephrectomy. An undetectable level of insulin was not consistent with insulinoma, making NICTH most likely (13, 20). Prior to the histopathologic diagnosis, metastasis of pulmonary adenocarcinoma or mammary carcinoma were considered potential causes of hypoglycemia given the patient's previous history of neoplasia and documentation in veterinary literature of IGF-2 secretion from these forms of neoplasia. This hypothesis would be unlikely as the patient's original neoplasms did not express an IGF-2 phenotype given the lack of previous hypoglycemia. Ultimately, a nephroblastoma with immunohistochemistry staining supporting IGF-2 expression was diagnosed and was determined to be the cause of hypoglycemia as the patient's blood glucose concentration improved almost immediately after nephrectomy. Moreover, >50% of the neoplastic cells were immunoreactive for IGF-2 immunohistochemistry staining.

Overproduction of IGF-2 has been described in pediatric human patients with Wilms' tumors (17) with increased IGF-2 expression in 50% of cases. This was the basis for selecting immunohistochemistry staining for IGF-2 in this case. Immunohistochemistry staining revealed that the neoplastic cells within the kidney were immunoreactive for IGF-2. The negative control stained appropriately, and this staining was not considered a feature of normal renal tubular cells. IGF-2 is synthesized in the glomerular and peritubular vasculature and interstitium and is largely produced by podocytes. Expression is also identified in normal renal tubules. Due to the IGF-2 expression in the normal renal tubules, it was difficult to establish if this expression was part of the oncogenetic mechanism or is a retained differentiation of the tubular origin of the neoplasm (16). Given the resolution of hypoglycemia after nephrectomy, tumor IGF-2 upregulation is considered the causative mechanism in this case. Insulin-like growth factor-2 causes hypoglycemia *via* multiple pathways including inhibition of hepatic glucose output, activation of insulin-receptors, and inhibition of gluconeogenesis, ketogenesis, and glycogenolysis. This hormone also stimulates glucose uptake in the muscle, inhibits lipolysis, and suppresses IGF-1, growth hormone, and glucagon production (17). In future cases, quantification of IGF-2 secretion within the blood may be considered, but is not currently commercially available for veterinary patients.

Treatment for hypoglycemia secondary to IGF-2 secretion is directed at controlling IGF-2 secretion (18). Hypoglycemia that resolves with surgical tumor resection has been documented in children with Wilms' tumors. Normoglycemia is maintained until tumor recurrence is reported (19). For humans, surgical treatment is considered standard within the United States and is followed by chemotherapy. Non-resectable tumors are addressed with a combination of medical treatment and radiation therapy.

Medical treatment of NICTH may include chemotherapy, frequent feedings, administration of IV dextrose, and glucagon administration. Of these medical treatments, response of NICTH to chemotherapy has generally been poor, whereas frequent feedings and administration of intravenous dextrose have been successful at controlling hypoglycemia in some cases (19). Continuous intravenous glucagon administration has also been described as a successful short-term, adjunctive therapy prior to surgery or as a palliative treatment. Glucagon acts by promoting glycogenolysis and gluconeogenesis, alongside inhibition of glycolysis and glycogenesis. A glucagon stimulation test is performed to predict the patient's response to the administration of glucagon and whether it will be efficacious. In humans, failure of response to the glucagon stimulation test indicates the presence of liver failure or hepatic glycogen depletion (16). Lack of response to glucagon in this case was unanticipated and may have been secondary to glycogen depletion due to potential chronicity of hypoglycemia, lean body condition, and the dog's active lifestyle leading to low glycogen stores. Exertional hypoglycemia has been documented in lean hunting dogs but is usually associated with extreme exercise and is transient (20). Glycogen depletion is suspected to be multi-factorial in this dog.

Additional agents that suppress insulin secretion such as octreotide, diazoxide, and calcium channel blockers are ineffective in IGF-2-producing tumors. Growth hormone and glucocorticoids have shown beneficial effects in humans with NICTH; growth hormone increases the rates of gluconeogenesis and glycolysis, while corticosteroids down-regulate the production of IGF-2 (13).

Interestingly, this patient displayed a hyperlactatemia and base deficit on presentation, although it is unclear what the underlying cause was. In non-diabetic patients, hypoglycemia can result in increased sensitivity to β adrenergic stimulation, which in turn can result in hyperlactatemia. Another potential cause of hyperlactatemia in this case would be type B1 hyperlactatemia secondary to neoplasia (21).

In human patients with nephroblastoma, prognostic factors for survival include histopathologic features (favorable or unfavorable as deemed by the presence of anaplasia) and clinical stages (I-V) (22). These prognostic factors have been adopted for use in canine patients. This patient would be considered to have Stage I disease with a favorable histopathological classification, although it is important to note that the lesion within the contralateral kidney was not sampled. If this lesion was consistent with metastasis, then this patient's disease would be considered Stage V. Median survival times of dogs with primary renal nephroblastoma reported in veterinary literature range from 4 to 25 months with a lower clinical stage and favorable histopathology associated with a longer median survival time (23, 24).

This case was unique for several reasons. The dog involved was a medium-sized breed, geriatric animal while historically, dogs diagnosed with nephroblastoma are young, large-breed dogs (1). In addition, this dog experienced severe hypoglycemia refractory to glucagon administration, which has not been previously reported in the veterinary literature. The presence of IGF-2 immunostaining within 50% of the cells supports IGF-2 as a cause of severe hypoglycemia, which has only been previously hypothesized. Finally, this case highlights the importance of urgent surgical tumor resection for animals refractory to medical management of hypoglycemia secondary to NICTH.

## 4. Conclusion

To the best of our knowledge, this is the first case report detailing the treatment of severe, refractory hypoglycemia in a dog with a renal nephroblastoma that was able to be resolved with nephrectomy. This is also the first case report detailing IGF-2 expression in a canine renal nephroblastoma as the likely cause of hypoglycemia. Medical management was insufficient to control severe hypoglycemia in this dog, supporting the notion that surgical resection is the preferential treatment for patients with NICTH.

## Data availability statement

The original contributions presented in the study are included in the article/supplementary material, further inquiries can be directed to the corresponding author.

## Ethics statement

Ethical review and approval was not required for the animal study because this is a case report and no experimental procedures were performed. The owner consented to all diagnostics and treatment interventions performed.

## Author contributions

BL and EM managed this patient prior to and after surgery. AB performed the surgery. LT was the oncologist managing the patient's long-term care. PS drafted the initial manuscript. LR and RF provided histopathology images and legends. All authors critically reviewed the manuscript and provided revisions. All authors contributed to the manuscript's final revision and approved the submitted manuscript.

## Footnotes

^1^SNAP 4DX, IDEXX, Westbrook, ME.

^2^INSULIN PANEL-CANINE/EQUINE, IDEXX, Westbrook, ME.
